# Factors influencing single young adults’ intentions to accept AI virtual companion apps based on the theory of emotional design

**DOI:** 10.3389/fpsyg.2026.1827571

**Published:** 2026-05-29

**Authors:** Ting Liu, Yue Sun, Zheng-Qi Wei, Ting-Yun Lo

**Affiliations:** School of Design, Fujian University of Technology, Fuzhou, China

**Keywords:** AI virtual companion apps, behavioral intention, structural equation modeling, theory of emotional design, young adults

## Abstract

**Background:**

AI virtual companion apps are increasingly used by single young adults as emotionally responsive media, yet acceptance studies often treat them as ordinary AI services or chatbots. This study examines how emotional design influences single young adults’ intention to accept AI virtual companion apps.

**Methods:**

Drawing on emotional design theory, technology acceptance research, and human–AI companionship literature, a conceptual model was developed in which emotional design affects behavioral intention directly and indirectly through user experience. Emotional design was operationalized through four AI-companion-specific dimensions: interaction quality, visual appeal, perceived personalization, and intelligent adaptability. Measurement items were adapted from validated scales and refined through expert interviews. A survey was conducted with 635 Chinese single young adults who had used AI virtual companion apps for at least 1 month. Data were analyzed using reliability analysis, exploratory and confirmatory factor analyses, correlation analysis, structural equation modeling, and bootstrap mediation testing.

**Results:**

The model showed adequate fit (CMIN/DF = 1.279; GFI = 0.980; AGFI = 0.971; IFI = 0.995; RMSEA = 0.021; CFI = 0.995; TLI = 0.994). Emotional design positively predicted user experience (*β* = 0.447, *p* < 0.001) and behavioral intention (*β* = 0.377, *p* < 0.001), while user experience also positively predicted behavioral intention (*β* = 0.310, *p* < 0.001). Intelligent adaptability had the strongest correlation with acceptance intention (*r* = 0.408, *p* < 0.01), followed by perceived personalization (*r* = 0.371, *p* < 0.01). User experience partially mediated the relationship between emotional design and behavioral intention, with an indirect effect of 0.139 and a total effect of 0.515.

**Conclusion:**

Intelligent adaptability and personalization appear especially important for the acceptance of AI virtual companion apps, while interaction quality and visual appeal support users’ initial and ongoing experiences. These findings clarify how AI-specific emotional design features become behaviorally meaningful through user experience and contribute to media psychology and human–AI interaction research.

## 1 Introduction

In contemporary society, loneliness among single young adults is shaped by urbanization, social isolation, limited offline interaction, and unmet emotional needs; studies in China show that urbanization is associated with both emotional and social loneliness, while younger adults may experience higher emotional loneliness under conditions of reduced social contact (Chen and Gong, 2022; Teater et al., 2021). AI virtual companion apps are intelligent conversational systems designed to provide social, emotional, and practical companionship through natural-language interaction, personalization, memory, avatars, and emotionally responsive dialogue (Chou et al., 2025; Strohmann et al., 2023). For this reason, AI virtual companions have emerged as a possible supplementary channel of emotional support because they are continuously available, personalized, and able to make users feel heard (De Freitas et al., 2024; Zhang and Li, 2025).

However, AI virtual companion apps differ from conventional AI tools because they combine technological functionality with emotionally meaningful interaction, relationship-building, and identity-related self-expression (Chaturvedi et al., 2023; Kouros and Papa, 2024). Their acceptance is therefore not determined only by efficiency or perceived usefulness, but also by emotional attachment, perceived empathy, customization, privacy concerns, and the perceived authenticity of companionship (Irfan et al., 2024; Zhang and Li, 2025). This gap raises an important question about how design elements influence users’ experience of companionship and their willingness to continue using such apps. Emotional design theory proposes that products should not only fulfill functional needs but also evoke affective resonance and create meaningful experiences; Norman’s visceral, behavioral, and reflective levels provide a useful starting point for understanding immediate aesthetic impressions, interaction quality, and the symbolic or personal meanings users attach to AI companions (Norman, 2007). Recent AI-companion research similarly suggests that continuity, contextual memory, self-expression, and emotional responsiveness may shape attachment and continued engagement (Liu et al., 2026; Mukherjee et al., 2026).

Previous studies on AI virtual companions have mainly examined technical implementation, functional design, ethical concerns, social presence, and attachment-like interaction (Boine, 2023; Chaturvedi et al., 2023; Kouros and Papa, 2024; Strohmann et al., 2023). Fewer studies have systematically explained how emotional design shapes acceptance intention among single young adults, especially within the Chinese cultural context (Liu et al., 2026; Xie and Wang, 2024). This context is important because cultural expectations regarding intimacy, privacy, stigma, and digital companionship may influence whether users treat AI companions as acceptable sources of support or as problematic substitutes for human contact (Meier et al., 2020; Zhang and Li, 2025).

To strengthen the theoretical positioning, this study treats AI virtual companion apps as relational media rather than as ordinary productivity-oriented AI systems (Chaturvedi et al., 2023, 2024). In technology acceptance research, behavioral intention is commonly explained through perceived usefulness, ease of use, hedonic motivation, trust, and perceived risk (Békés et al., 2025; Chen et al., 2020). Studies on wearable and AI-related technologies further show that acceptance depends not only on technical performance but also on enjoyment, trust, privacy risk, cultural background, and contextual evaluations (Meier et al., 2020; Peng et al., 2022; Shrivastava, 2025). From a broader disruptive-technology perspective, users’ and organizations’ capacity to absorb, interpret, and integrate novel digital technologies also affects how AI systems become embedded in practice (Kastelli et al., 2024; Păvăloaia and Necula, 2023). These perspectives help position the present model within media psychology, technology acceptance, and human–AI interaction research.

Accordingly, the novelty of this study lies in three aspects. First, it integrates emotional design theory with technology acceptance and digital companionship literature to explain acceptance as an experiential and relational process (Chaturvedi et al., 2023; Strohmann et al., 2023). Second, it clarifies how Norman’s emotional design levels can be translated into AI companion-specific dimensions (Norman, 2007). Third, it empirically tests whether user experience mediates the effect of emotional design on behavioral intention in a relatively large sample of Chinese single young adults who have actual experience using AI companion apps (Liu et al., 2026; Xie and Wang, 2024).

As such, the following core questions were proposed: According to the theory of emotional design, what are the factors influencing single young adults’ intentions to accept AI virtual companions? This question was further divided into the following sub-questions:

(1) How do different dimensions of emotional design (i.e., interaction quality, visual appeal, perceived personalization, and intelligent adaptability) influence single young adults’ intentions to accept AI virtual companions?
(2) Does user experience mediate between emotional design and acceptance intention?
(3) How should these relationships be interpreted within the demographic and cultural context of Chinese single young adults?

**Figure 1:**
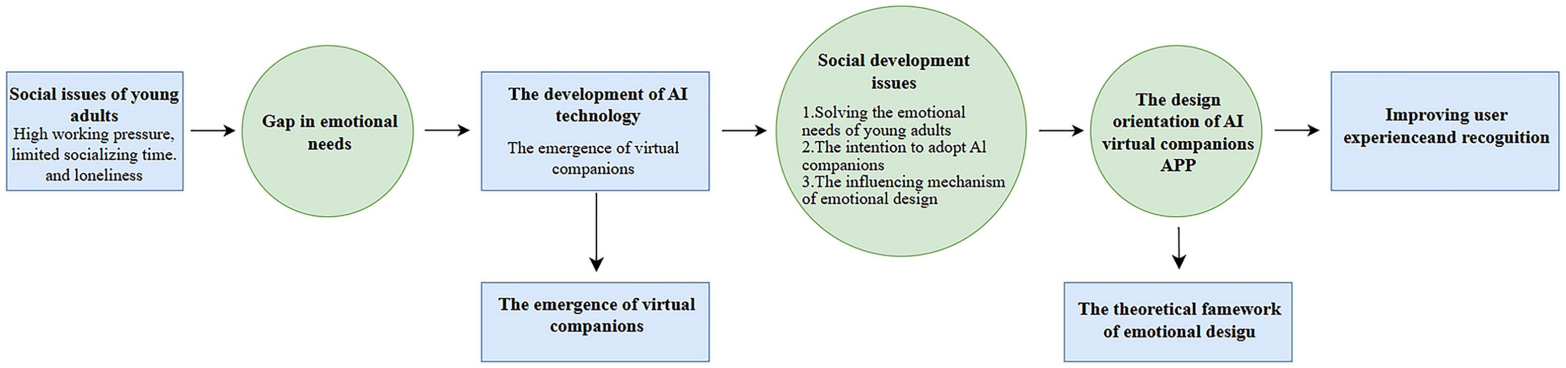
Research association.

The purpose was to construct an analytical framework integrating emotional design theory, technology acceptance research, human–AI interaction, and digital companionship literature to elucidate the mechanisms influencing single young adults’ intentions to accept AI virtual companions (Figure 1; Chaturvedi et al., 2023; Norman, 2007; Strohmann et al., 2023). As previous studies have emphasized, understanding users’ emotional needs is pivotal for the development of AI products, especially when such products are designed for companionship, emotional expression, and loneliness-related support (De Freitas et al., 2024; Xie and Wang, 2024). This research further examines whether and how that claim applies to AI virtual companion apps.

Furthermore, the potential value of AI virtual companions as emotionally supportive tools has become increasingly visible as public awareness of mental health and loneliness has grown (De Freitas et al., 2024; Savic, 2024). Nevertheless, this study does not treat AI companion apps as clinical interventions or as substitutes for human relationships; rather, existing research suggests that AI companions should be understood as supplementary tools whose benefits must be balanced against risks such as emotional dependency, privacy concerns, and weakened human connection (Jacobs, 2024; Knox et al., 2025). Rather, it examines design-related acceptance mechanisms that may make such apps more or less acceptable as supplementary digital companions (Chaturvedi et al., 2024; Mukherjee et al., 2026).

**Figure 2:**
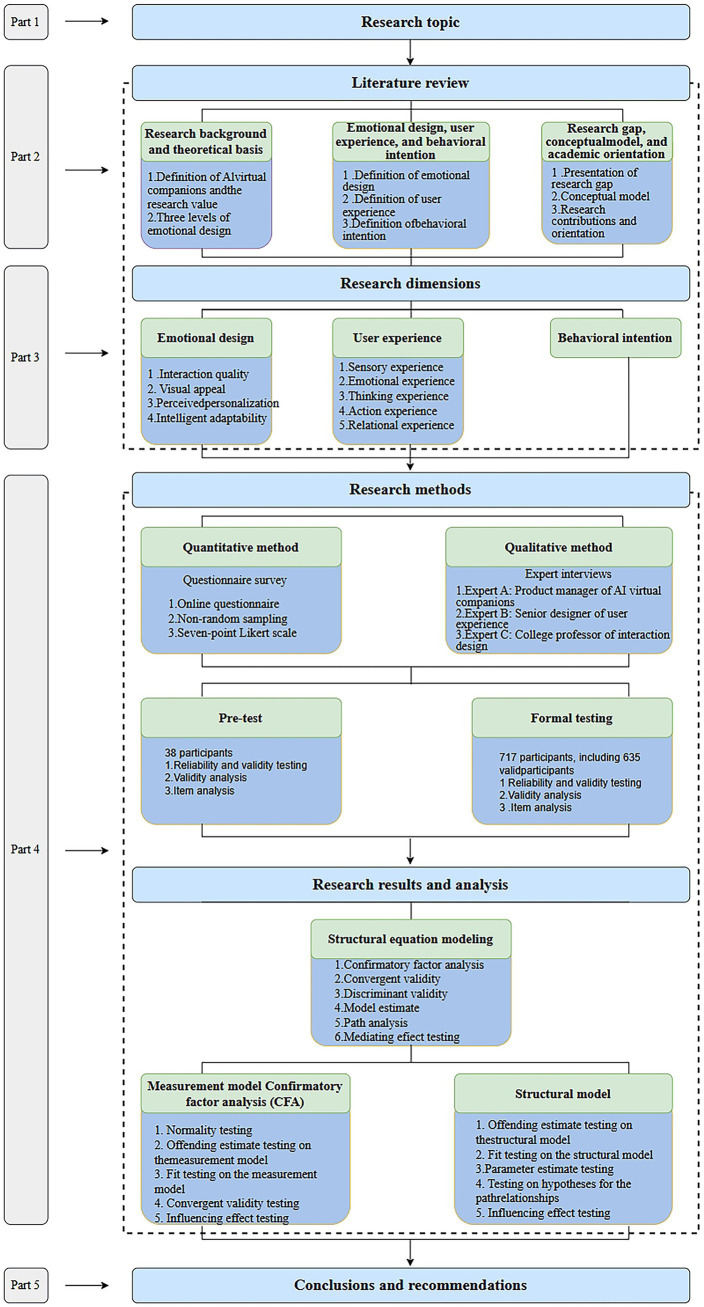
Research framework.

Specifically, the research data were collected through questionnaire surveys, the relationships among the variables were analyzed through structural equation modeling (SEM), and expert interviews were used to refine the measurement dimensions and enrich the interpretation of design implications; this mixed-method logic is consistent with studies that combine interviews, questionnaires, and SEM to build and validate human-centered technology acceptance models (Akram et al., 2024; Ullman and Bentler, 2012). The findings are expected to contribute to theory by connecting emotional design with AI companion acceptance, and to practice by providing empirically grounded design suggestions for more responsible and user-sensitive AI virtual companion apps (Anisha et al., 2024; Strohmann et al., 2023; Figure 2).

## 2 Literature review and hypothesis development

The evolution of artificial intelligence virtual companion apps has been rapid, moving from peripheral digital culture into everyday mediated life as conversational agents increasingly support social, emotional, and practical interaction rather than only task completion (Chou et al., 2025; Strohmann et al., 2023). The utilisation of these systems has evolved beyond their original purpose as mere tools for information retrieval or entertainment, with users increasingly treating them as emotionally responsive systems that simulate listening, memory, care, and companionship (Kouros and Papa, 2024; Savic, 2024). Recent studies indicate that individuals seek out AI companions not only because of their accessibility, but also because these systems can feel attentive, personalized, emotionally validating, and psychologically soothing during loneliness or relational stress (Chaturvedi et al., 2024; De Freitas et al., 2024; Lai et al., 2025). For single young adults, whose emotional lives are often shaped by mobility, unstable work trajectories, delayed marriage, and digitally mediated sociality, this promise may be especially compelling, although direct evidence on this exact subgroup remains limited (Liu et al., 2026; Xie and Wang, 2024). However, the notion that AI companions can possess emotional significance does not inherently explain why some users accept them while others remain sceptical; the more pressing theoretical question concerns which design qualities make an AI companion engaging and credible to interact with (Brandt and Wang, 2025; Mukherjee et al., 2026).

The present study approaches the question through emotional design. Emotional design theory offers a useful lens for understanding why users do not respond to products solely in instrumental terms (Norman, 2007). Norman argued that design functions at visceral, behavioral, and reflective levels, shaping immediate sensory impressions, perceived usability, and the meanings users attach to an object in relation to the self (Norman, 2007). In the context of AI companionship, these levels can be translated into the interface through which users first encounter the system, the interaction process through which companionship becomes experientially available, and the longer-term perception that the companion knows, understands, and responds to the user as a distinct person (Liu et al., 2026; Strohmann et al., 2023).

Research on virtual companionship lends support to this perspective. A growing body of scholarship has demonstrated that companionship technologies become persuasive when they combine social presence, relational continuity, and adaptive responsiveness. Users have been shown to be more likely to remain engaged when interactions feel natural, when the system appears to be emotionally available, and when the companion can sustain a coherent persona over time (Merrill et al., 2022; Strohmann et al., 2023; Xie and Pentina, 2022). Conversely, when app updates disrupt the perceived identity of an AI companion, users may experience disappointment, distrust, and even a sense of relational rupture. This suggests that acceptance cannot be reduced to functional efficiency alone (De Freitas et al., 2024). The extant literature on the subject suggests that, in the context of AI companions, design exerts a significant influence on the emotional credibility of the user experience.

Research on virtual companionship supports this perspective. A growing body of scholarship shows that companionship technologies become persuasive when they combine social presence, relational continuity, adaptive responsiveness, and coherent persona design (Chaves and Gerosa, 2021; Strohmann et al., 2023). Users are more likely to remain engaged when interactions feel natural, emotionally available, and stable over time (Brandt and Wang, 2025; De Freitas et al., 2024). Conversely, when app changes or shutdowns disrupt the perceived identity or availability of an AI companion, users may experience disappointment, distrust, grief, or relational rupture (Banks, 2024; Lee et al., 2026). This suggests that acceptance cannot be reduced to functional efficiency alone; in AI companions, design strongly shapes the emotional credibility of the user experience (Chaturvedi et al., 2024; Magni et al., 2021).

Technology acceptance literature provides a complementary perspective. Classic and extended acceptance models emphasize that behavioral intention emerges from users’ evaluations of perceived usefulness, ease of use, enjoyment, trust, and risk (Chen et al., 2002; Wang and Yu, 2026). For AI-related technologies, these evaluations are often intensified because users must decide whether the system is not only useful but also acceptable, safe, trustworthy, and meaningful in everyday life (Bai and Yang, 2025; Fu et al., 2025). These insights are relevant to AI virtual companions because companionship apps are evaluated through affective and relational criteria as much as instrumental ones (Chaturvedi et al., 2024; Mukherjee et al., 2026).

The present study therefore uses emotional design as the primary theoretical lens while positioning user experience as the mechanism through which design qualities are translated into behavioral intention. This approach helps distinguish AI virtual companions from other AI systems. A search engine, recommendation system, or productivity chatbot may be accepted mainly because it provides accurate information or saves time, whereas an AI virtual companion is accepted when the user perceives continuity, attentiveness, emotional appropriateness, and the possibility of relationship-like interaction (Kouros and Papa, 2024; Strohmann et al., 2023). Acceptance in this setting is thus simultaneously technological, experiential, and relational (Banks, 2024; Niess and Woźniak, 2020).

Guided by emotional design theory and prior studies on virtual companions, this study conceptualizes emotional design through four interrelated dimensions: interaction quality, visual appeal, perceived personalization, and intelligent adaptability. Visual appeal corresponds primarily to the visceral level because it shapes immediate sensory impressions and affective readiness (Norman, 2007). Interaction quality corresponds primarily to the behavioral level because it concerns fluency, coherence, response speed, and perceived ease of use during interaction (Noor et al., 2022; Strohmann et al., 2023). Perceived personalization corresponds primarily to the reflective level because it concerns whether users feel recognized as distinct individuals with their own histories, preferences, and emotional needs (Kouros and Papa, 2024; Liu et al., 2026). Intelligent adaptability is not treated as a purely emotional dimension in itself; rather, it is conceptualized as an AI-mediated capability that supports behavioral and reflective emotional design by allowing the companion to learn from prior interactions and adjust responses over time (Chaves and Gerosa, 2021; Gentile et al., 2007). This clarification is important because the study does not equate technical sophistication with emotional design; intelligent adaptability becomes part of emotional design only when users experience it as responsiveness, continuity, and personally meaningful care (Banks, 2024; Chaturvedi et al., 2024).

Visual appeal corresponds most directly to the visceral level. Before a longer-term relationship-like interaction can develop, users encounter an interface, avatar, color system, tone of presentation, and broader aesthetic atmosphere; these cues shape first impressions and influence whether the system appears inviting, trustworthy, and emotionally compatible (Cao et al., 2025; Norman, 2007). Although visual design alone is not sufficient for sustained engagement, it often serves as a critical threshold condition that determines whether users find a companion app sufficiently approachable to explore (Savic, 2024; Tao et al., 2025). In this sense, visual appeal is not merely decorative; it establishes the symbolic and affective frame within which subsequent interaction is interpreted (Segaran et al., 2021).

Perceived personalization is associated mainly with the reflective level because it concerns whether the user feels recognized as a particular person rather than treated as a generic user. A companion app becomes more than a general chatbot when it can remember preferences, adapt communicative style, refer to prior conversations, and respond in ways that feel tailored rather than standardized (Chaturvedi et al., 2024; Strohmann et al., 2023). This is important because users evaluate companion technologies not only according to function but also according to whether the system affirms personal relevance, identity exploration, and emotional uniqueness (Kouros and Papa, 2024; Ma et al., 2026). For single young adults, who may seek validation, recognition, and conversational continuity, perceived personalization is likely to be especially consequential (Liu et al., 2026).

Intelligent adaptability extends these ideas into the specifically AI-driven capacity of the system to learn from interaction over time. Although it is grounded in technological capability, it has emotional significance when adaptive responses are perceived as sensitive to the user’s changing mood, habits, and needs (Li, 2025). In contrast to static digital products, AI virtual companions may adjust conversational strategies, infer patterns, and modulate responses on the basis of accumulated interaction histories (Xi and Wang, 2025). This feature is important because companionship is inherently temporal: a companion that does not evolve may be perceived as repetitive or superficial, whereas a companion that adapts can create an impression of relational continuity (Banks, 2024; Lee et al., 2026). Recent scholarship on AI companionship and human–AI attachment highlights continuity, learning, and adaptive responsiveness as central to sustained engagement (Liu et al., 2026). Therefore, intelligent adaptability is included in the model as an AI-specific emotional design capability, while its technological nature and ethical implications are acknowledged (Knox et al., 2025).

### 2.1 Emotional design and user experience

The field of user experience occupies a central position in the study of digital media adoption, given that design influences behavior through users’ dynamic encounters with digital systems (Guo et al., 2025; Wei et al., 2025). The effects of the system are realised through the user’s lived encounter with it. In emotional design theory, product meaning emerges when formal and functional properties are translated into affective and cognitive responses during use (Norman, 2007). This logic is particularly relevant to AI virtual companions, where acceptance depends less on objective technical capacity alone than on perceived interaction quality, comfort, immersion, and emotional gratification (Chaturvedi et al., 2023; Mukherjee et al., 2026).

The extant research supports the hypothesis that emotionally oriented design is closely related to user experience. In companion technologies and social chatbots, users report more positive experiences when systems display social presence, responsive dialogue, emotional availability, and continuity across interactions (De Freitas et al., 2024; Kouros and Papa, 2024; Strohmann et al., 2023). Research on emotionally attuned AI systems also suggests that conversational design can create perceived support, emotional safety, and reflective engagement beyond basic usability (Shi et al., 2026; Zhu et al., 2026). More recent analyses of AI companions further suggest that adaptive and personalized features strengthen satisfaction, perceived depth of interaction, and relationship-like engagement (Brandt and Wang, 2025; Ma et al., 2026).

The four dimensions identified in this study can each be understood as pathways through which emotional design shapes experience. Interaction quality reduces friction and supports conversational flow (Noor et al., 2022). Visual appeal influences affective readiness and initial attraction to the product (Cao et al., 2025). Perceived personalisation reinforces the perception that the system responds to the individual user rather than to an abstract user category (Kouros and Papa, 2024; Pan et al., 2025). Intelligent adaptability fosters continuity and novelty, thereby enabling a dynamic relationship that transcends scripted interactions (Liu et al., 2026; Gentile et al., 2007). Taken together, these dimensions suggest that emotional design should exert a positive influence on user experience in the context of AI virtual companion apps.

H1. Emotional design can positively influence user experience.

### 2.2 Emotional design and behavioral intention

The term “behavioral intention” refers to a user’s propensity to adopt, continue using, recommend, or pay for a technology. In technology acceptance and HCI research, behavioral intention is commonly treated as a proximal predictor of actual technology use (Alanazi et al., 2025; Salifu et al., 2024). However, intention is not formed only through rational cost–benefit assessment; trust, risk, hedonic motivation, and emotional satisfaction also shape users’ willingness to adopt digital systems (Chen et al., 2020; Wang and Yu, 2026). This is especially pronounced in affectively charged technologies, where engagement depends on whether the medium appears to align with users’ psychological and emotional needs (Merrill et al., 2022; Mukherjee et al., 2026). This is the basis upon which emotional design may influence behavioral intention both directly and indirectly.

A substantial body of research supports the expectation of such a relationship. Research on virtual companions shows that users are attracted to systems that exhibit social presence, emotional accessibility, warmth, and relational coherence (Chaturvedi et al., 2024; Merrill et al., 2022; Strohmann et al., 2023). Research on conversational AI further indicates that loneliness, mind perception, anthropomorphic cues, and parasocial or companionship-like processes can increase willingness to use such systems because they may be perceived as emotionally safer and more supportive interactional spaces (De Freitas et al., 2024; Lai et al., 2025; Liang et al., 2025). In summary, design can influence behavioral intention before deep attachment is established because users form early assessments of a system’s emotional appropriateness and appeal (Brandt and Wang, 2025).

This direct effect is theoretically plausible in the case of AI virtual companion applications. The aesthetic appeal and emotional responsiveness of an app can prompt immediate trial intention, while personalisation and adaptability can reinforce continuance intention over time (Merrill et al., 2022; Mukherjee et al., 2026). In both cases, emotional design functions not merely as decoration but also as a signal of relational potential (Chaturvedi et al., 2023). A design perceived as cold, rigid, generic, or incoherently adaptive may suppress willingness to engage, whereas a design perceived as attentive and emotionally congruent can invite use by reducing psychological distance (Brandt and Wang, 2025; Liang et al., 2025). Consequently, emotional design is expected to exert a favourable influence on behavioral intention toward AI virtual companion applications.

H2. Emotional design can positively influence behavioral intention.

### 2.3 User experience and behavioral intention

The concept of emotional design pertains to the offerings of the system, whereas user experience relates to the user’s feelings and interpretations during engagement. This distinction is significant because technologies are not accepted merely because they possess certain features, but because those features are transformed into meaningful experiences (Deng et al., 2010). User experience has been widely shown to predict satisfaction, loyalty, and continued use across digital contexts (Cheng and Jiang, 2020; Jeon, 2024). In AI-based systems, where uncertainty and novelty may initially produce hesitation, positive experiences are especially important because they reduce ambiguity and support sustained use (Kim et al., 2025; Xu et al., 2024).

Research on social chatbots and companion technologies indicates a similar trend. Attachment-based analyses of Replika users show that when users perceive the system as emotionally supportive, encouraging, and psychologically secure, they may sustain interaction and develop attachment to the platform (Xie and Pentina, 2022). Studies of Replika and social chatbots further show that acceptance, non-judgmental interaction, self-disclosure, and affective value can support relationship development and continued engagement (Brandtzaeg et al., 2022; Skjuve et al., 2021). Research into AI companionship also demonstrates links between social presence, comfort, emotional resonance, and stronger acceptance or recommendation intention (Guingrich and Graziano, n.d.; Merrill et al., 2022). Research on virtual companionship indicates that the experiential dimension of use is inseparable from decisions about whether a companion is worth returning to, recommending, or integrating into daily routines (Chaturvedi et al., 2024; Savic, 2024).

The influence of user experience is likely to be especially pronounced for single young adults, since the value of an AI companion is likely to be judged through emotionally laden criteria such as relief, recognition, intimacy, and conversational ease (Kouros and Papa, 2024; Zhang et al., 2025). A technically proficient system that fails to create engagement or personal connection is unlikely to gain lasting acceptance, whereas an experience perceived as warm, smooth, and personally meaningful may directly translate into stronger behavioral intention (Kim et al., 2025; Mukherjee et al., 2026). On this basis, the following hypothesis is advanced.

H3. User experience can positively influence behavioral intention.

### 2.4 The mediating role of user experience

The preceding arguments suggest not only separate relationships among emotional design, user experience, and behavioral intention, but also a more integrated process. Emotional design may not influence intention only through direct evaluation; rather, users are more likely to accept a technology when design features are transformed into satisfying and meaningful experiences (Deng et al., 2010; Kim et al., 2025). This mediating logic is consistent with emotional design theory, which argues that design produces behavioral consequences through affective response, cognitive interpretation, and lived interaction (Norman, 2007).

The validity of this assertion is further substantiated by empirical studies in related domains. Research on AI companions, virtual relationships, and emotionally intelligent systems shows that design features become influential when they enhance perceived empathy, social presence, warmth, trust, and experiential fit (Cai et al., 2024; Kim et al., 2025; Strohmann et al., 2023). Research on digital loneliness and AI companionship likewise indicates that users do not simply respond to isolated functions; instead, they respond to the perceived experience of being accompanied, understood, or psychologically comforted (De Freitas et al., 2024; Jacobs, 2024). In this sense, user experience is not a secondary outcome, but the mechanism through which emotional design becomes behaviorally consequential (Puertas et al., 2024).

This mediation pathway appears especially plausible for AI virtual companion applications because the product category itself is built around experience. Users do not adopt a companion app only to improve task efficiency; they adopt it because the interaction may feel emotionally fulfilling, comforting, or personally significant (Skjuve et al., 2021; Xie and Pentina, 2022). Consequently, emotional design should cultivate stronger behavioral intention at least partly by enhancing user experience. The final hypothesis therefore states the following.

H4. User experience mediates between emotional design and behavioral intention.

To make the derivation of the hypotheses explicit, the literature review was translated into the model through a three-step logic. First, emotional design theory explains how visceral, behavioral, and reflective design cues shape users’ affective and cognitive evaluations. Second, technology acceptance research explains how such evaluations become behavioral intention when users judge a technology to be valuable, acceptable, and easy to integrate into everyday life. Third, AI companionship literature shows that social presence, relational continuity, personalization, and adaptive responsiveness are especially salient for companion apps. On this basis, H1 links emotional design to user experience, H2 links emotional design directly to behavioral intention, H3 links user experience to behavioral intention, and H4 tests user experience as the mediating mechanism between design and intention.

**Figure 3:**
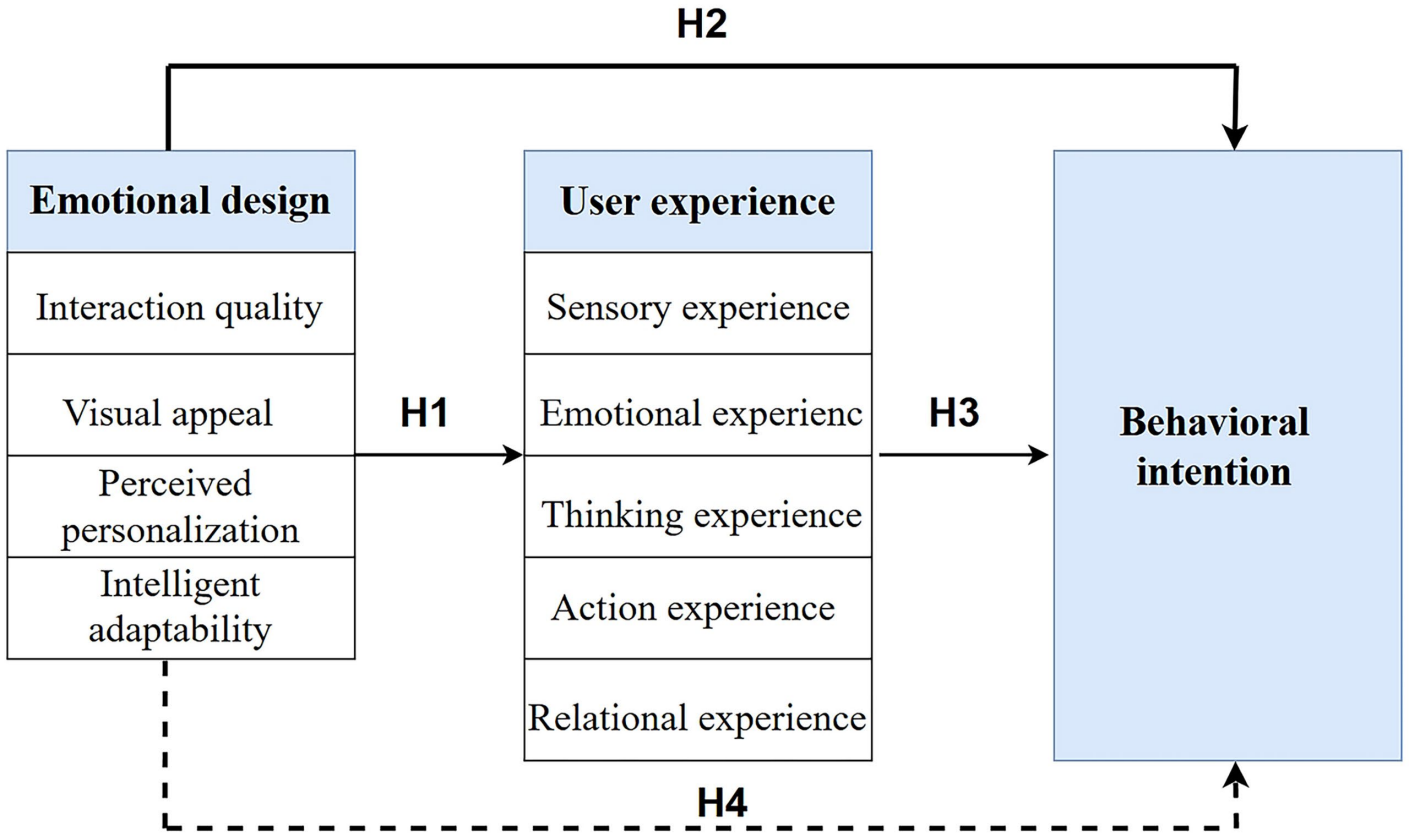
Research hypotheses.

To sum up, the research hypotheses are as displayed in Figure 3.

## 3 Research model

This section is repositioned as a main topic because the research model is the bridge between the literature review and the empirical design. It synthesizes the theoretical logic into a structural model for SEM testing rather than functioning as a subtopic of the literature review.

In accordance with the aforementioned reasoning, the present study proposes a model in which emotional design functions as the independent variable, user experience functions as the mediating variable, and behavioral intention functions as the dependent variable. Emotional design is represented through four dimensions particularly relevant to AI virtual companion apps: interaction quality, visual appeal, perceived personalization, and intelligent adaptability. User experience refers to the overall experiential evaluation generated through contact with the system, while behavioral intention refers to willingness to accept, continue using, recommend, or pay for related services.

**Figure 4:**
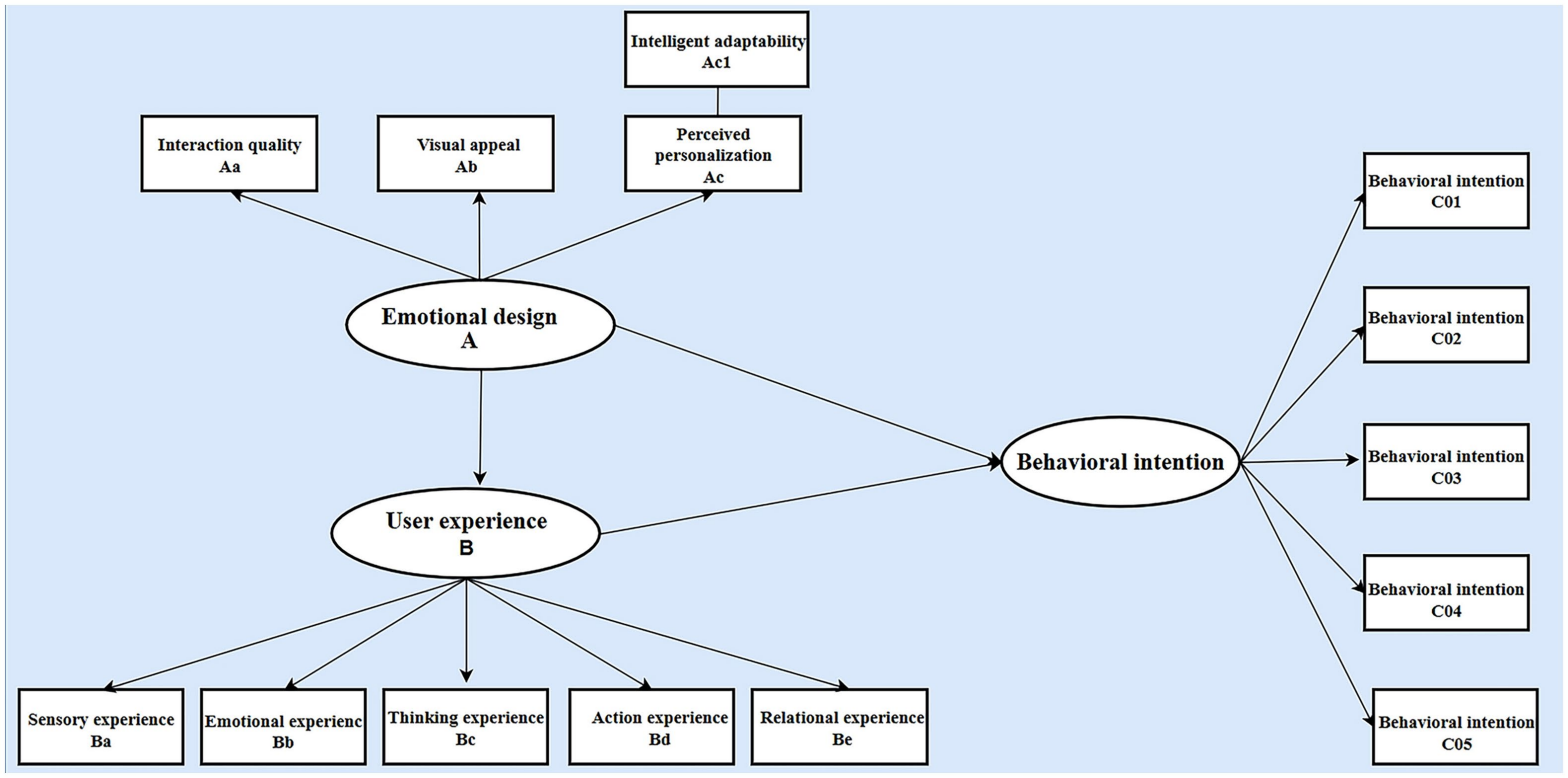
The theoretical model for structural equation modeling (SEM).

The model posits that emotional design influences behavioral intention in two ways. One pathway is direct, as users may form immediate judgments about whether a companion app appears emotionally suitable and worth adopting. The other pathway is indirect, because emotionally attuned design may enhance user experience, and a more positive experience may subsequently strengthen behavioral intention. In this manner, the model explains acceptance not as a simple response to technical functionality, but as a psychologically mediated process in which design, experience, and intention are sequentially linked (Figure 4).

## 4 Research design and methods

### 4.1 Expert interviews

Expert interviews were conducted to comprehensively understand the factors influencing single young adults’ intentions to accept AI virtual companions. Three experts were involved in the expert review. They were selected through purposive sampling because each had 5–10 years of directly relevant experience in one of the following areas: AI virtual companion product development, user experience and emotional interaction design, or academic research on human-computer interaction and emotional design. The purpose of the expert review was not to generate statistically generalizable findings, but to refine the construct definitions, improve item wording, check the practical relevance of the model, and identify ethical and contextual issues that might not be sufficiently visible from literature review alone. In this exploratory design stage, three experts were considered sufficient because they represented complementary product, design, and academic perspectives, and their comments converged around the key dimensions subsequently used in the questionnaire. The experts interviewed are presented in Table 1.

**Table 1:** Experts interviewed.

No.	Interviewee	Title	Expertise	Relevant experience (5–10 years)
E1	Wen◯◯	Product manager of AI virtual companions	Product planning, algorithm implementation, and commercialization	Responsible for the product roadmap and market promotion of multiple virtual companion APPs.
E2	Yang◯◯	Senior designer of user experience	Design of social robots and emotional interaction interfaces	Leading the interface and character development of social robots and virtual agents.
E3	Jiang◯◯	College professor of interaction design	Human-computer interaction and emotional design theory	Published over 20 papers regarding HCI and affective computing, and presided over national-level research projects.

The interviews were conducted in a semi-structured form, with a session of approximately 30–45 min for each expert and an interview outline pre-formulated by the research team. The outline covered emotional design, personalization and intelligent adaptability, technology perception and usage barriers, ethical and social acceptance, usage scenarios and cultural differences, and future product trends (Table 2). The interviews were carried out either in person or via video conference, and prior consent for audio recording and citation was obtained from all participants. The collected information was used to refine questionnaire wording, supplement ethical considerations, and enrich the context for subsequent discussion.

**Table 2:** Expert interview item analysis.

Module	Sub-dimension	Question No.	Interview question (semi-structured guidance)	Purpose and explanation
I. Emotional design	Visceral level	Q1-1	What key features do you think an AI virtual companion should possess in terms of appearance or interface to create an immediate good impression on single young adults?	To deepen the definition of “visual appeal” and capture design guidelines.
		Q1-2	What experience of success or failure can you share to avoid the “uncanny valley” effect?	To gather feasible design boundaries and risks.
	Behavioral level	Q1-3	During interactions, which details can best enhance the “dialogue fluency” and the “sense of being understood”? Please provide real-life examples.	To clarify the evaluation dimensions of “interaction quality.”
		Q1-4	How do you measure the impact of AI response speed and semantic accuracy on emotional experience?	To provide measurement ideas for questionnaire indicators.
	Reflective level	Q1-5	How can AI companions provide users with emotional support or self-recognition through symbols, stories, or a sense of ritual?	To explore mechanisms for deep emotional resonance.
II. Personalization and intelligent adaptability	Data-driven learning	Q2-1	What user data do you think the system should record and learn from to truly provide a personalized companionship experience?	To delineate the scope of data collection.
		Q2-2	When an AI companion “misremembers” or “forgets,” what are the typical emotional responses of users?	To reveal the risks of adaptive failures.
	Contextual adaptation	Q2-3	How should an AI companion distinguish different emotional states (e.g., loneliness, anxiety, and excitement) in its response strategies?	To refine the logic of emotion recognition and feedback.
III. Technology perception and usage barriers	Perceived usefulness/ease of use	Q3-1	What specific pain points do single young adults most expect AI companions to address in daily life?	To clarify the scenarios of “perceived usefulness.”
		Q3-2	In actual deployments, which technical flaws are most likely to cause loss of users? How should they be improved in priority?	To rank improvement priorities.
IV. Ethical and social acceptance	Privacy security	Q4-1	What kind of design do you think should be made at the product, protocol, or operational level to make users believe that their chat content is absolutely confidential?	To correspond with the “trustworthiness” item in the questionnaire.
	Emotional dependency	Q4-2	How do you judge whether users are overly dependent on AI companions or not? What preventive mechanisms can be designed?	To enrich ethical indicators.
	Social image	Q4-3	How do you view the “stigma surrounding virtual companions”? What positioning or promotional strategies can help reduce negative labels?	To guide the setting of contextual variables.
V. Usage scenarios and cultural differences	Scenario triggers	Q5-1	In your observations, what time, device, or location do users interact with AI companions most frequently?	To define “high-frequency usage scenarios.”
	Cultural factors	Q5-2	Which kind of emotional design element should be particularly adjusted in different cultural or regional backgrounds? Please provide examples.	To provide a basis for cross-cultural extrapolation.
VI. Future trends and business strategies	Product iteration	Q6-1	What are the development trends in emotional design for AI companions over the next 3–5 years?	A predictive question for discussion.
	Business models	Q6-2	Among paid subscriptions, value-added services, or advertising placements, which model is more acceptable to single young adults?	To link to the derivative variables of “behavioral intention.”

### 4.2 Questionnaire design

To ensure validity and reliability, the final questionnaire was designed on the basis of validated scales from the existing literature, expert interview findings, and the specific characteristics of AI virtual companion apps. The questionnaire focused on the factors influencing single young adults’ intentions to accept AI virtual companions, especially the four emotional design dimensions identified above. A seven-point Likert scale was used to quantify participants’ responses, ranging from 1 = “strongly disagree” to 7 = “strongly agree.” This approach allowed the study to capture nuanced evaluations of each factor and to conduct subsequent quantitative analysis.

The questionnaire structure was divided into two parts. The first part collected basic information, including age, gender, education, occupation, income, and primary purpose of using AI virtual companions. The second part measured emotional design, user experience, and behavioral intention. All measurement items were adapted from prior validated scales rather than used as entirely new, unvalidated items. The wording was modified to fit the AI virtual companion context; for example, general interaction or technology acceptance items were rephrased to refer specifically to the virtual companion’s dialogue fluency, emotional response, memory, personalization, and adaptive behavior. The expert interviews were used to assess face validity, contextual appropriateness, and whether the items adequately captured the visceral, behavioral, reflective, and AI-adaptive characteristics of emotional design. The item wording and scale sources are reported in Table 3.

**Table 3:** Behavior measurement scale.

Variable	Item	Content	References
Interaction quality	IQ1	I feel a smooth interaction with the virtual companion.	Recommended scale sources: Borsci et al. (2022), BUS-15/Chatbot Usability Scale—conversational efficiency, functionality and time response for chatbot interaction quality; Chen et al. (2022), AICSQ—AI chatbot service quality, especially responsiveness/understanding/personalization; Pelau et al. (2021)—interaction quality and empathy in AI acceptance. IQ5 may also draw on Schmidmaier et al. (2024), PETS—emotional responsiveness/understanding.
	IQ2	The virtual companion responds quickly.	
	IQ3	I feel that the content of conversations with the virtual companion is natural.	
	IQ4	The virtual companion can understand my needs accurately.	
	IQ5	The virtual companion’s emotional responses make me feel understood.	
Visual appeal	VA1	The virtual companion’s appearance design is highly appealing to me.	Recommended scale sources: Moshagen and Thielsch (2010), VisAWI—visual aesthetics dimensions of simplicity, diversity, colorfulness and craftsmanship; Lavie and Tractinsky (2004)—perceived visual aesthetics of websites, classical and expressive aesthetics. Suitable for avatar appearance, style, interface aesthetics, color and visual delight.
	VA2	I like the virtual companion’s image and style.	
	VA3	The virtual companion’s interface design is aesthetically pleasing and easy to understand.	
	VA4	The virtual companion’s visual effects bring me a sense of delight.	
	VA5	I’m satisfied with the color and overall appearance design of the virtual companion.	
Perceived personalization	PP1	The virtual companion can accurately recognize the emotions I express.	Recommended scale sources: Schmidmaier et al. (2024), Perceived Empathy of Technology Scale (PETS)—emotional responsiveness and understanding/trust items such as understanding needs/goals and responding to mental/emotional states; Fan and Poole (2006)—personalization in information systems: what/to whom/who personalizes; Chen et al. (2022), AICSQ—personalized/relevant AI chatbot service. This construct is best named perceived empathic personalization.
	PP2	The virtual companion can make appropriate responses based on my emotional state.	
	PP3	I perceive an emotional resonance from the virtual companion.	
	PP4	The virtual companion’s responses make me feel understood.	
	PP5	I feel an emotional comfort through interactions with the virtual companion.	
Intelligent adaptability	IA1	The virtual companion can adjust its functions based on my past behaviors.	Recommended scale sources: Fan and Poole (2006)—personalization implementation choices: content, interface, functionality and channel; Chen et al. (2022), AICSQ—AI chatbot service quality attributes and personalized/relevant service; Chen et al. (2023)—AI chatbot service quality attributes affecting perceived value, trust, satisfaction and loyalty. Adapt for behavior history, emotional state, personality/preferences and tailored suggestions.
	IA2	The virtual companion can tailor its interaction style according to my emotional state and needs.	
	IA3	The virtual companion has adapted to my personality and preferences.	
	IA4	I feel that the virtual companion can adjust its response approach at appropriate moments.	
	IA5	The virtual companion can provide content and suggestions aligning with my specific needs.	
Sensory experience	SE1	The virtual companion’s visual effects bring me a sense of pleasure.	Recommended scale sources: Brakus et al. (2009)—sensory dimension of brand experience; Schmitt (1999a, 1999b)—SENSE experiential module; Hone and Graham (2000), SASSI—subjective assessment of speech system interfaces for voice/speech interaction, likeability and response qualities. Suitable for visual effects and auditory/voice experience.
	SE2	The virtual companion’s voice sounds natural.	
	SE3	I enjoy the visual effects during my interactions with the virtual companion.	
	SE4	The virtual companion’s voice offers me a great auditory experience.	
	SE5	The virtual companion’s design fully meets my expectations in both visual and auditory aspects.	
Emotional experience	EX1	I feel happy in the interaction with the virtual companion.	Recommended scale sources: Brakus et al. (2009)—affective dimension of brand experience; Schmitt (1999a, 1999b)—FEEL experiential module; Schmidmaier et al. (2024), PETS—emotional responsiveness of technology. For loneliness relief, De Freitas et al. (2024/2025) on AI companions reducing loneliness can be used as contextual support.
	EX2	The virtual companion makes me feel cared for.	
	EX3	I’m satisfied with the virtual companion’s emotional responses.	
	EX4	The virtual companion’s interactions provide me with emotional comfort.	
	EX5	My interactions with the virtual companion effectively alleviate my feelings of loneliness.	
Thinking experience	TE1	I feel excited and curious about the interaction with the virtual companion.	Recommended scale sources: Brakus et al. (2009)—intellectual dimension of brand experience; Schmitt (1999a, 1999b)—THINK experiential module, curiosity, creative cognition and problem-solving; Laugwitz et al. (2008), UEQ—stimulation/novelty items may supplement curiosity and cognitive novelty.
	TE2	The content provided by the virtual companion sparks more thought and contemplation in me.	
	TE3	I often receive creative suggestions when interacting with the virtual companion.	
	TE4	The interactions with the virtual companion bring me new cognitive experiences.	
	TE5	I’m truly amazed by the virtual companion’s intelligence level and thinking ability.	
Action experience	AE1	I feel that the virtual companion’s way of interaction makes me more actively engaged in the process.	Recommended scale sources: Brakus et al. (2009)—behavioral dimension of brand experience; Schmitt (1999a, 1999b)—ACT experiential module, physical behaviors, lifestyles and interactions. Suitable for active engagement, proactivity, daily tasks and activity participation.
	AE2	The interaction with the virtual companion motivates me to participate in more activities.	
	AE3	The virtual companion’s feedback enhances my intention to engage in physical activities or tasks.	
	AE4	I think the interactions with the virtual companion make me more proactive.	
	AE5	The functions offered by the virtual companion prompt me to be more involved in daily tasks.	
Relational experience	RE1	I feel a sense of belonging when interacting with the virtual companion.	Recommended scale sources: Schmitt (1999a, 1999b)—RELATE experiential module; Nysveen et al. (2013)—relational/social experience in service brand experience; Lee and Robbins (1995)—Social Connectedness Scale for belonging/connectedness; McMillan and Chavis (1986)—sense of community theory for belonging, membership and shared emotional connection.
	RE2	The virtual companion makes me feel that I’m a part of a certain community.	
	RE3	I think the virtual companion enables me to resonate more with others.	
	RE4	My interactions with the virtual companion evoke a feeling of cultural recognition in me.	
	RE5	The virtual companion’s social function makes me feel more connected when I’m alone.	
Behavioral intention	BI1	I have the intention to continue using the virtual companion.	Recommended scale sources: Venkatesh et al. (2003), UTAUT—behavioral intention to use technology; Bhattacherjee (2001), IS continuance intention—continued use; Zeithaml et al. (1996)—behavioral consequences of service quality, including recommendation, positive word of mouth and purchase/repurchase intention.
	BI2	I plan to purchase relevant services associated with the virtual companion in the future.	
	BI3	I’d like to recommend the virtual companion to my family or friends.	
	BI4	I would consider buying or trying new virtual companion products if any.	
	BI5	I’d like to use the virtual companion in my daily life.	

Several procedures were used to strengthen measurement transparency. First, item sources were documented for each construct in Table 3. Second, the research team localized item wording for Chinese single young adult users while preserving the conceptual meaning of the original scales. Third, three experts in AI product development, user experience design, and interaction design reviewed the measurement dimensions and suggested wording refinements. Fourth, the formal dataset was evaluated through reliability analysis, exploratory factor analysis, confirmatory factor analysis, convergent validity, discriminant validity, and common method bias checks. Thus, the measurement model was not assumed to be valid solely because the items came from prior studies; it was empirically reassessed in the present sample.

### 4.3 Data collection

#### 4.3.1 Sampling and participants

The questionnaire survey was conducted from November 2025 to February 2026, with data collected online. The questionnaire link was disseminated through social media platforms such as WeChat, Weibo, and RedNote, as well as relevant interest-based communities. Snowball sampling was used to broaden sample coverage. Screening questions were placed at the beginning of the questionnaire to ensure that participants matched the target population: they had to be 18–35 years old, single, and have at least 1 month of AI virtual companion use experience. The 1-month threshold was used to ensure that participants were familiar enough with AI virtual companions to provide meaningful evaluations of the questionnaire items.

Because the study relied on online recruitment and snowball sampling, the sample should be interpreted as a group of digitally active Chinese single young adults with prior AI companion experience, rather than as a nationally representative sample of all young adults or all single people in China. This sampling strategy improved the relevance of responses to the product category, but it also limited generalizability. Users without prior AI companion experience, users outside China, and young adults who are less active on social media may evaluate emotional design and AI companionship differently.

Each eligible participant completing the questionnaire was provided with a digital red envelope with a certain amount to raise the effective collection rate. A total of 717 copies of the questionnaire were distributed. Consequently, 686 copies were collected. Among them, 635 copies were valid, excluding invalid questionnaires with over-short completion times (less than one-third of the average completion time), obvious response laws (e.g., selecting the same options consecutively), and missing critical information. The sample size far exceeded 10 times the total number of measurement items (30) in the model, meeting the basic requirements for SEM analysis. The basic demographic characteristics of the participants are displayed in Table 4.

**Table 4:** Demographic characteristics of the samples.

Item	Option	Frequency	Percentage	Cumulative percentage
Gender	Male	329	51.8	51.8
	Female	306	48.2	100
Age	18–24 years old	298	46.9	46.9
	25–35 years old	337	53.1	100
Education background	Higher vocational school, senior high school, and inferior level	187	29.4	29.4
	Undergraduate or junior college	255	40.2	69.6
	Master	124	19.5	89.1
	PhD and superior level	69	10.9	100
Primary purposes of using virtual companions	Emotional support	155	24.4	24.4
	Information inquiry/search	161	25.4	49.8
	Social entertainment	150	23.6	73.4
	Learning or work assistance	169	26.6	100
Occupation	Military/civil servant	134	21.1	21.1
	Agriculture/farming	122	19.2	40.3
	Industrial/blue-collar worker	121	19.1	59.4
	Business	136	21.4	80.8
	N/A	122	19.2	100
Monthly income	Below RMB 10,000 Yuan	125	19.7	19.7
	RMB 10,000–20,000 Yuan	191	30.1	49.8
	RMB 20,000–30,000 Yuan	207	32.6	82.4
	More than RMB 30,000 Yuan	112	17.6	100

As observed in Table 4, there were 329 male participants (51.8%) and 306 female participants (48.2%). Regarding age, 298 participants (46.9%) were 18–24 years old, and 337 participants (53.1%) were aged 25–35. In terms of educational background, 187 participants (29.4%) held a diploma of higher vocational school, high school, or lower levels of school; 255 participants (40.2%) had a bachelor’s degree or junior college diploma; 124 participants (19.5%) possessed a master’s degree; and 69 participants (10.9%) had a doctoral or higher degree. AI virtual companions were primarily used for emotional support (155 participants, 24.4%), information inquiry/search (161 participants, 25.4%), social entertainment (150 participants, 23.6%), and learning or work assistance (169 participants, 26.6%). The participants’ occupations were military/civil servant (134 participants, 21.1%), agriculture/farming (122 participants, 19.2%), industrial/blue-collar worker (121 participants, 19.1%), business (136 participants, 21.4%), and N/A (122 participants, 19.2%), respectively. The distribution of monthly income was as follows: below RMB 10,000 Yuan (125 participants, 19.7%), RMB 10,000–20,000 Yuan (191 participants, 30.1%), RMB 20,000–30,000 Yuan (207 participants, 32.6%), and more than RMB 30,000 Yuan (112 participants, 17.6%).

## 5 Data analysis

### 5.1 Expert interview data analysis

To validate and supplement the model constructed in this research, the interview data from three experts was analyzed qualitatively using a six-step thematic analysis method: (1) familiarizing with the data; (2) initial coding; (3) searching for topics; (4) examining and approving the topics; (5) defining and naming; (6) writing the report. NVivo 14 was employed to perform open coding on the transcribed texts, generating 126 initial nodes. After axial coding, five core topics were obtained, as shown in Table 5.

**Table 5:** Topics in the expert interviews—Connotations, typical quotations, and model mapping.

Topic	Connotation (key point)	Typical quotation (quoted from what the expert said)	Corresponding relation with the research model
Visual presentation	- Initial attraction and uninstallation threshold - Avoidance of the “uncanny valley” effect and prevention of homogenization - Needed customizable options such as skins/themes	“Young users will not give you a chance to ‘warm up slowly’; if it does not appeal at first glance, they’ll uninstall it.” said E2.	Corresponding to visual appeal (visceral level); directly influencing user experience.
Interactive intelligence	- Natural language understanding and conversation coherence - Emotional recognition and empathetic response - Cross-session learning and memory	“Users will soon realize it’s just an advanced voice assistant if an AI can only respond mechanically.” said E1.	Corresponding to interaction quality; intersecting with perceived personalization to jointly influence user experience and acceptance intention
Personalized perception	- Memorizing personal details and recalling them at the right time - Dynamically adjusting tone/rhythm - Ensuring transparent and controllable data collection	“I’d be instantly touched if an AI can remember my complaints yesterday and proactively ask me about that today.” said E3.	Corresponding to perceived personalization/intelligent adaptability; positively influencing user experience and acceptance intention
Ethical acceptability	- Encryption of chat data and storage location - Psychological risks of excessive dependence - Social stigma and positioning strategies	“What users are worried about is not the AI itself, but rather who might witness their vulnerabilities.” said E2.	Serving as a moderating variable (trust) influencing the strength of the path from emotional design to behavioral intention
Usage scenario	- High-frequency periods: late at night, during commuting, and in fragmented rest intervals - Typical emotions: loneliness, stress, boredom - Cultural differences: Japanese and Korean > European and American > Chinese	“Many people do not lack friends; rather, they cannot find someone to chat with at 2 a.m.” said E1.	Serving as a control variable (contextual fit) for adjusting the overall model error

### 5.2 Reliability analysis

The measurement model was assessed in several steps. Internal consistency was examined using Cronbach’s alpha and Corrected Item-Total Correlation (CITC). Exploratory factor analysis was then used to examine the underlying dimensionality of the emotional design and user experience scales, with KMO and Bartlett’s test used to confirm suitability for factor analysis. Confirmatory factor analysis further evaluated the measurement model through standardized factor loadings, Composite Reliability (CR), Average Variance Extracted (AVE), and discriminant validity. In addition, Harman’s single-factor test was used as an initial diagnostic for common method bias, because all constructs were measured through the same questionnaire at one point in time.

### 5.3 Measurement model assessment

Reliability and validity analyses were conducted using data selected from the scales. Cronbach’s alpha was employed to assess the data reliability. In reliability analysis, a Cronbach’s alpha coefficient exceeding 0.7 generally indicates a high reliability of the questionnaire, thereby justifying further in-depth analysis of the questionnaire’s associated aspects. As can be seen in the tables below, Cronbach’s alpha coefficients for all dimensions and the overall questionnaire exceeded 0.7; the Corrected Item-Total Correlation (CITC) values were all above 0.4; the Cronbach’s alpha coefficients calculated after item deletion were consistently lower than the original coefficients for their respective dimensions. These data demonstrated a high reliability of the overall questionnaire, without items requiring deletion (Tables 6–8).

**Table 6:** Reliability analysis of the emotional design scale.

Dimension	Sub-dimension	Item	Corrected item-total correlation	Cronbach’s alpha if item deleted	Cronbach’s alpha	Overall Cronbach’s alpha
Emotional design	Interaction quality	IQ1	0.722	0.874	0.893	0.932
		IQ2	0.742	0.869		
		IQ3	0.74	0.87		
		IQ4	0.75	0.868		
		IQ5	0.738	0.87		
	Visual appeal	VA1	0.753	0.858	0.888	
		VA2	0.725	0.865		
		VA3	0.725	0.865		
		VA4	0.735	0.862		
		VA5	0.702	0.87		
	Perceived personalization	PP1	0.741	0.86	0.888	
		PP2	0.727	0.864		
		PP3	0.726	0.864		
		PP4	0.719	0.865		
		PP5	0.724	0.864		
	Intelligent adaptability	IA1	0.73	0.867	0.890	
		IA2	0.718	0.87		
		IA3	0.76	0.86		
		IA4	0.724	0.869		
		IA5	0.731	0.867		

**Table 7:** Reliability analysis of the user experience scale.

Dimension	Sub-dimension	Item	Corrected item-total correlation	Cronbach’s alpha if item deleted	Cronbach’s alpha	Overall Cronbach’s alpha
User Experience	Sensory experience	SE1	0.723	0.861	0.886	0.952
		SE2	0.733	0.859		
		SE3	0.722	0.862		
		SE4	0.722	0.862		
		SE5	0.719	0.862		
	Emotional experience	EX1	0.733	0.865	0.889	
		EX2	0.744	0.863		
		EX3	0.748	0.862		
		EX4	0.724	0.867		
		EX5	0.706	0.871		
	Thinking experience	TE1	0.7	0.871	0.888	
		TE2	0.729	0.864		
		TE3	0.748	0.86		
		TE4	0.74	0.862		
		TE5	0.726	0.865		
	Action experience	AE1	0.731	0.868	0.891	
		AE2	0.744	0.866		
		AE3	0.741	0.866		
		AE4	0.741	0.866		
		AE5	0.715	0.872		
	Relational experience	RE1	0.746	0.85	0.882	
		RE2	0.719	0.857		
		RE3	0.718	0.857		
		RE4	0.71	0.859		
		RE5	0.694	0.863		

**Table 8:** Reliability analysis of the behavioral intention scale.

Dimension	Item	Corrected item-total correlation	Cronbach’s alpha if item deleted	Cronbach’s alpha
Behavioral intention	BI1	0.72	0.851	0.879
	BI2	0.704	0.855	
	BI3	0.702	0.856	
	BI4	0.731	0.848	
	BI5	0.699	0.856	

### 5.4 Validity analysis

Factor analysis was made to assess the validity of the questionnaire. In validity analysis, a KMO measure keeping above 0.7 generally signifies the suitability for factor analysis of the questionnaire. As presented in Table 9, all KMO values derived from the test exceeded 0.7; and Bartlett’s test of sphericity yielded a significance level (Sig.) of 0.000, showing statistical significance at the 0.001 level. These results confirmed the appropriateness of conducting factor analysis.

**Table 9:** KMO and Bartlett’s test results (emotional design).

KMO measure of sampling adequacy		0.949
Bartlett’s test of sphericity	Approximate chi-square	7,442.058
	Degrees of freedom (DOF)	190
	Significance level	0.000

Through further in-depth analysis, Table 10 was obtained. As shown, the total variance explained by the factors extracted from the emotional design scale was 69.640%. This denoted that the factors had good explanatory power and that the four extracted factors could comprehensively retain the original data information. Meanwhile, the variance extracted by the first-factor loading without rotation was 43.696% (below 45%). This suggested that the questionnaire did not have serious common method biases.

**Table 10:** Total variance explained.

Component	Initial eigenvalue			Extraction sums of squared loadings			Rotation sums of squared loadings		
	Total	Variance percentage	Cumulative %	Total	Variance percentage	Cumulative %	Total	Variance percentage	Cumulative %
1	8.739	43.696	43.696	8.739	43.696	43.696	3.515	17.573	17.573
2	1.842	9.212	52.908	1.842	9.212	52.908	3.508	17.540	35.113
3	1.817	9.084	61.992	1.817	9.084	61.992	3.471	17.357	52.470
4	1.530	7.648	69.640	1.530	7.648	69.640	3.434	17.170	69.640
5	0.467	2.337	71.977						
6	0.460	2.302	74.279						
7	0.456	2.281	76.560						
8	0.432	2.158	78.718						
9	0.430	2.149	80.867						
10	0.404	2.019	82.886						
11	0.397	1.985	84.871						
12	0.392	1.958	86.830						
13	0.366	1.829	88.659						
14	0.357	1.784	90.443						
15	0.340	1.698	92.141						
16	0.332	1.658	93.799						
17	0.324	1.618	95.416						
18	0.311	1.557	96.973						
19	0.311	1.555	98.528						
20	0.294	1.472	100.000						

As observed from the factor loadings in Table 11, all items in the emotional design scale fell within their corresponding pre-set dimensions. This implied that the questionnaire had good construct validity and that the data obtained from it could be used for further analysis. The entire questionnaire demonstrated high reliability and validity and could be employed for the research analysis (Tables 11, 12).

**Table 11:** Rotated component matrix.

Item	Component			
	1	2	3	4
IQ1	0.768			
IQ2	0.771			
IQ3	0.768			
IQ4	0.776			
IQ5	0.786			
VA1				0.772
VA2				0.752
VA3				0.794
VA4				0.751
VA5				0.743
PP1			0.771	
PP2			0.794	
PP3			0.749	
PP4			0.763	
PP5			0.752	
IA1		0.770		
IA2		0.759		
IA3		0.802		
IA4		0.773		
IA5		0.769		

**Table 12:** KMO and Bartlett’s test results (user experience).

KMO measure of sampling adequacy		0.964
Bartlett’s test of sphericity	Approximate chi-square	9,819.015
	Degrees of freedom (DOF)	300
	Significance level	0.000

Through further in-depth analysis, Table 13 was obtained. As displayed, the total variance explained by the factors extracted from the user experience scale was 69.284%. Namely, the five extracted factors had good explanatory power and could fully retain the original data information. Meanwhile, the variance extracted by the first-factor loading without rotation was 46.492% (below 50%). This explained that the questionnaire did not have serious common method biases.

**Table 13:** Total variance explained.

Component	Initial eigenvalue			Extraction sums of squared loadings			Rotation sums of squared loadings		
	Total	Variance percentage	Cumulative %	Total	Variance percentage	Cumulative %	Total	Variance percentage	Cumulative %
1	11.623	46.492	46.492	11.623	46.492	46.492	3.544	14.176	14.176
2	1.663	6.653	53.144	1.663	6.653	53.144	3.531	14.126	28.301
3	1.467	5.870	59.014	1.467	5.870	59.014	3.466	13.866	42.167
4	1.312	5.249	64.263	1.312	5.249	64.263	3.392	13.566	55.734
5	1.255	5.021	69.284	1.255	5.021	69.284	3.388	13.550	69.284
6	0.552	2.208	71.491						
7	0.517	2.068	73.560						
8	0.502	2.008	75.568						
9	0.466	1.865	77.433						
10	0.437	1.747	79.179						
11	0.425	1.700	80.879						
12	0.417	1.668	82.547						
13	0.404	1.616	84.163						
14	0.386	1.544	85.707						
15	0.375	1.501	87.208						
16	0.369	1.474	88.682						
17	0.353	1.411	90.093						
18	0.350	1.399	91.492						
19	0.338	1.352	92.844						
20	0.327	1.309	94.152						
21	0.320	1.282	95.434						
22	0.307	1.230	96.664						
23	0.290	1.161	97.824						
24	0.287	1.146	98.971						
25	0.257	1.029	100.000						

According to the factor loadings in Table 14, all items in the user experience scale were within their corresponding pre-set dimensions. This signified that the questionnaire had good construct validity and that the data obtained from it could be used for further analysis. In general, the questionnaire demonstrated high reliability and validity and could be used for the research analysis (Tables 14, 15).

**Table 14:** Rotated component matrix.

Item	Component				
	1	2	3	4	5
SE1			0.742		
SE2			0.729		
SE3			0.743		
SE4			0.697		
SE5			0.732		
EX1	0.733				
EX2	0.753				
EX3	0.757				
EX4	0.738				
EX5	0.7				
TE1				0.709	
TE2				0.74	
TE3				0.749	
TE4				0.694	
TE5				0.696	
AE1		0.708			
AE2		0.743			
AE3		0.756			
AE4		0.745			
AE5		0.724			
RE1					0.739
RE2					0.731
RE3					0.747
RE4					0.673
RE5					0.708

**Table 15:** KMO and Bartlett’s test results (behavioral intention).

KMO measure of sampling adequacy		0.883
Bartlett’s test of sphericity	Approximate chi-square	1,509.915
	Degrees of freedom (DOF)	10
	Significance level	0.000

Through further in-depth analysis, the total variance explained by the factor extracted from the behavioral intention scale was obtained to be 56.443%. This indicated that the factor extracted had good explanatory power and could comprehensively retain the original data information (Table 16).

**Table 16:** Total variance explained.

Component	Initial eigenvalue			Extraction sums of squared loadings		
	Total	Variance percentage	Cumulative %	Total	Variance percentage	Cumulative %
1	5.644	56.443	56.443	5.644	56.443	56.443
2	0.695	6.955	63.398			
3	0.557	5.568	68.965			
4	0.543	5.427	74.392			
5	0.478	4.784	79.176			
6	0.466	4.656	83.832			
7	0.454	4.545	88.377			
8	0.437	4.365	92.742			
9	0.384	3.839	96.581			
10	0.342	3.419	100.000			

### 5.5 Descriptive statistical analysis

The variables were also analyzed by descriptive statistical means. As presented in Tables 17–19, the absolute kurtosis was all less than 10 and the absolute skewness was all less than 3. Therefore, the variables could be considered as in normal distribution.

**Table 17:** Descriptive statistics of the emotional design scale.

Item	Number of cases	Minimum	Maximum	Mean	Standard deviation	Skewness	Kurtosis
Interaction quality	635	1.2	6.8	4.0784	1.53041	−0.178	−1.257
Visual appeal	635	1.0	7.0	3.9735	1.49647	−0.046	−1.169
Perceived personalization	635	1.2	6.8	4.0214	1.51078	−0.062	−1.221
Intelligent adaptability	635	1.0	6.8	4.1244	1.53782	−0.137	−1.242
Emotional design	635	1.65	6.3	3.8982	1.1628	−0.018	−1.142

Within the emotional design scale, the mean value was 4.0784 for interaction quality, 3.9735 for visual appeal, 4.0214 for perceived personalization, and 4.1244 for intelligent adaptability; the overall mean value for emotional design was 3.8982.

**Table 18:** Descriptive statistics of the user experience scale.

Item	Number of cases	Minimum	Maximum	Mean	Standard deviation	Skewness	Kurtosis
Sensory experience	635	1.4	6.8	4.0948	1.50893	−0.088	−1.249
Emotional experience	635	1.0	7.0	4.0280	1.52225	−0.081	−1.219
Thinking experience	635	1.0	6.8	4.0674	1.49362	−0.19	−1.211
Action experience	635	1.2	7.0	4.1855	1.52217	−0.167	−1.155
Relational experience	635	1.0	7.0	4.1524	1.48088	−0.125	−1.137
User experience	635	1.8	6.28	3.9175	1.1454	0.112	−1.201

Within the user experience scale, the mean value was 4.0948 for sensory experience, 4.0280 for emotional experience, 4.0674 for thinking experience, 4.1855 for action experience, and 4.1524 for relational experience; the overall mean value for user experience was 3.9175.

**Table 19:** Descriptive statistics of the behavioral intention scale.

Item	Number of cases	Minimum	Maximum	Mean	Standard deviation	Skewness	Kurtosis
BI1	635	1	7	4.15	1.741	−0.103	−0.937
BI2	635	1	7	4.11	1.787	0.005	−0.97
BI3	635	1	7	4.13	1.790	−0.063	−0.998
BI4	635	1	7	4.23	1.827	−0.148	−1.058
BI5	635	1	7	4.10	1.772	−0.079	−0.986
Behavioral intention	635	1.4	6.8	4.142	1.46442	−0.153	−1.159

Within the consumer behavioral intention scale, the mean value was 4.15 for behavioral intention 1, 4.11 for behavioral intention 2, 4.13 for behavioral intention 3, 4.23 for behavioral intention 4, and 4.10 for behavioral intention 5; and the overall mean value for behavioral intention was 4.142.

### 5.6 Correlation analysis

Pearson correlation analysis was conducted to investigate whether there were significant correlations among emotional design, user experience, and behavioral intention. Among them, emotional design comprised interaction quality, visual appeal, perceived personalization, and intelligent adaptability; user experience encompassed sensory experience, emotional experience, thinking experience, action experience, and relational experience. As illustrated in Table 20, the significances of correlations between the variables were all less than 0.01, indicating the presence of significant correlations. Moreover, the correlation coefficients revealed that there were markedly positive correlations among the dimensions.

**Table 20:** Correlation analysis.

Item	Behavioral intention
Interaction quality	0.285
Visual appeal	0.303
Perceived personalization	0.371
Intelligent adaptability	0.408
Sensory experience	0.360
Emotional experience	0.325
Thinking experience	0.392
Action experience	0.301
Relational experience	0.345

As unveiled in Table 20:

(1) The correlation coefficient between behavioral intention and interaction quality was 0.285, with a significance level of 0.01, indicating a significantly positive correlation between behavioral intention and interaction quality;
(2) The correlation coefficient between behavioral intention and visual appeal was 0.303, with a significance level of 0.01, suggesting a markedly positive correlation between behavioral intention and visual appeal;
(3) The correlation coefficient between behavioral intention and perceived personalization was 0.371, with a significance level of 0.01, demonstrating a notably positive correlation between behavioral intention and perceived personalization;
(4) The correlation coefficient between behavioral intention and intelligent adaptability was 0.408, with a significance level of 0.01, showing a markedly positive correlation between behavioral intention and intelligent adaptability;
(5) The correlation coefficient between behavioral intention and sensory experience was 0.360, with a significance level of 0.01, reflecting a substantially positive correlation between behavioral intention and sensory experience;
(6) The correlation coefficient between behavioral intention and emotional experience was 0.325, with a significance level of 0.01, implying a prominently positive correlation between behavioral intention and emotional experience;
(7) The correlation coefficient between behavioral intention and thinking experience was 0.392, with a significance level of 0.01, signifying a notably positive correlation between behavioral intention and thinking experience;
(8) The correlation coefficient between behavioral intention and action experience was 0.301, with a significance level of 0.01, showing a significantly positive correlation between behavioral intention and action experience;
(9) The correlation coefficient between behavioral intention and relational experience was 0.345, with a significance level of 0.01, reflecting a markedly positive correlation between behavioral intention and relational experience.

### 5.7 Confirmatory factor analysis

**Figure 5:**
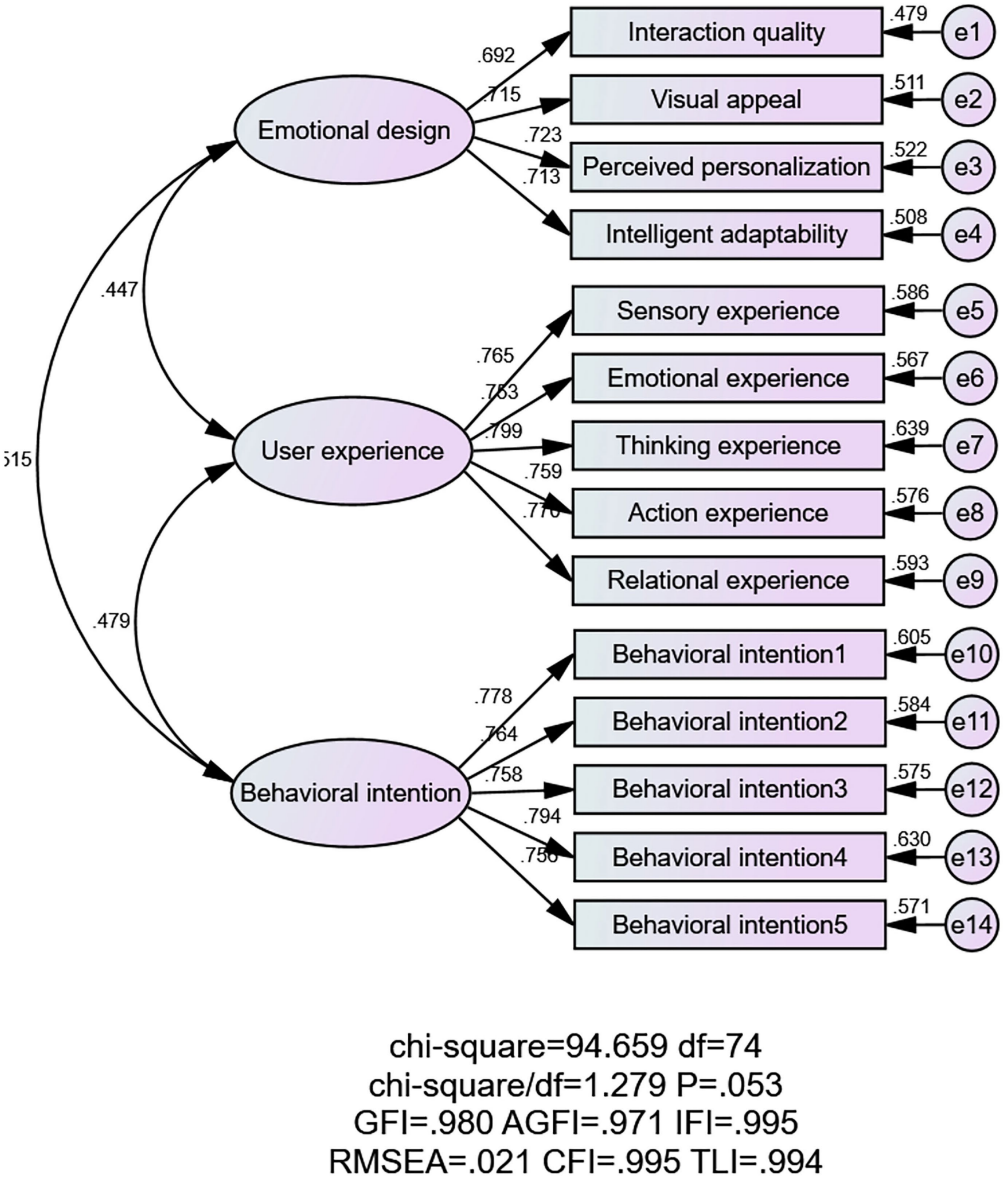
Diagram of confirmatory factor analysis.

Confirmatory Factor Analysis (CFA) was employed to further examine the measurement model. In the CFA, emotional design and user experience were treated as latent constructs represented by their corresponding first-order dimensions, while behavioral intention was represented by five observed items. Standardized factor loadings greater than 0.6, Composite Reliability (CR) greater than 0.7, and Average Variance Extracted (AVE) greater than 0.5 were used as criteria for acceptable convergent validity and construct reliability (Figure 5).

As observed from Table 21, the degrees of fitting of CFA met the ideal thresholds, proving the reliability of the analysis results.

**Table 21:** Confirmatory factor analysis.

Item	CMIN/DF	GFI	AGFI	IFI	RMSEA	CFI	TLI
Ideal value	≤3.00	≥0.90	≥0.90	≥0.90	≤0.08	≥0.90	≥0.90
Degree of fitting	1.279	0.980	0.971	0.995	0.021	0.995	0.994

As seen from Table 22, the data exhibited high composite reliability and construct validity as all the normalized factor loadings of the items and the CR and AVE values of the dimensions met the criteria.

**Table 22:** Normalized factor loading.

Item	Measurement item	Normalized factor loading	CR	AVE	AVE square root
Emotional design	Interaction quality	0.692	0.803	0.505	0.711
	Visual appeal	0.715			
	Perceived personalization	0.723			
	Intelligent adaptability	0.713			
User experience	Sensory experience	0.765	0.879	0.592	0.769
	Emotional experience	0.753			
	Thinking experience	0.799			
	Action experience	0.759			
	Relational experience	0.77			
Behavioral intention	BI1	0.778	0.879	0.593	0.770
	BI2	0.764			
	BI3	0.758			
	BI4	0.794			
	BI5	0.756			

Finally, the square roots of the AVEs for all dimensions were compared with the correlation coefficients between the dimensions. The results revealed that the square roots of AVEs for all dimensions were greater than the correlation coefficients between the dimensions. This indicated that the correlation within each dimension was stronger than the correlation between different dimensions, demonstrating good discriminant validity of the data. In summary, the data exhibited satisfactory reliability and validity, making further analysis suitable (Table 23).

**Table 23:** Discriminant validity.

Item	Emotional design	User experience	Behavioral intention
Emotional design	0.711		
User experience	0.447	0.769	
Behavioral intention	0.515	0.479	0.770

### 5.8 Structural model and hypothesis testing

**Figure 6:**
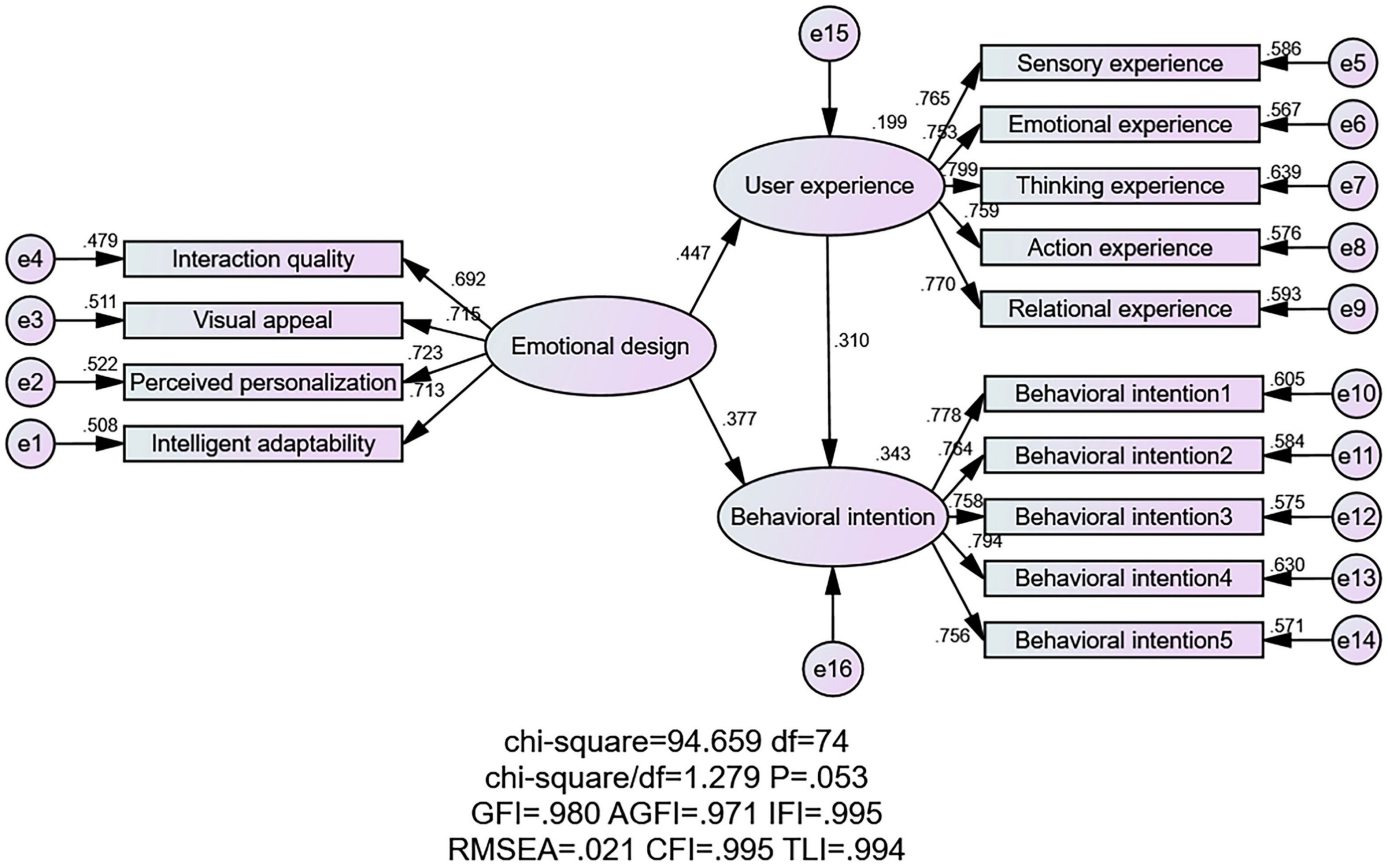
Diagram of path analysis.

The hypotheses developed in the literature review were tested in the structural model shown in Figure 6:

H1: Emotional design can positively influence user experience.

H2: Emotional design can positively influence behavioral intention.

H3: User experience can positively influence behavioral intention.

H4: User experience mediates between emotional design and behavioral intention.

Structural equations were employed to examine the path coefficients of the model. As a result, all degrees of fitting for the model achieved the ideal thresholds, evidencing a good fit of the model (Table 24).

**Table 24:** Path coefficients.

Item	CMIN/DF	GFI	AGFI	IFI	RMSEA	CFI	TLI
Ideal value	≤3.00	≥0.90	≥0.90	≥0.90	≤0.08	≥0.90	≥0.90
Degree of fitting	1.279	0.980	0.971	0.995	0.021	0.995	0.994

Further, the hypotheses were validated. As a result, the significance levels for all paths were less than 0.05, and H1, H2, and H3 were supported (Table 25).

**Table 25:** Verification results of the hypotheses.

Path	se	Estimate	S.E.	C.R.	*p*	Hypothesis
Emotional design → User experience	0.447	0.471	0.053	8.92	*	Supported
User experience → Behavioral intention	0.310	0.364	0.056	6.547	*	Supported
Emotional design → Behavioral intention	0.377	0.465	0.063	7.359	*	Supported

By 5,000 times of sampling, the mediating effect was tested using the PROCESS Bootstrap method and a 95% confidence interval. As shown in Table 26, the 95% CI for the indirect effect, direct effect, and total effect all did not encompass zero. Hence, the significance levels for these effects were below 0.05 and the hypotheses of these effects were markedly supported. User experience (mediating variable) partially mediated in the influence of emotional design (independent variable) on behavioral intention (dependent variable; Table 26).

**Table 26:** Mediating effect test results.

Item	Significance	Effect	95% CI		*p*-value	Conclusion
			Lower limit	Upper limit		
Emotional design → User experience → Behavioral intention	Indirect effect	0.139	0.098	0.188	0.000	H4 is supported
	Direct effect	0.377	0.284	0.463	0.000	
	Total effect	0.515	0.442	0.585	0.000	

## 6 Results and discussion

This research explored the design-related mechanisms influencing single young adults’ intentions to accept AI virtual companions based on emotional design theory. The four hypotheses were supported by the SEM results. The following discussion interprets the findings in relation to emotional design, technology acceptance, human–AI interaction, and digital companionship research, while also recognizing the cultural and methodological boundaries of the study.

### 6.1 Theoretical contribution and positioning

The first theoretical contribution is the integration of emotional design theory with technology acceptance and digital companionship research. Prior acceptance studies have often emphasized usefulness, ease of use, hedonic motivation, trust, and privacy risk (Magni et al., 2021). These factors remain relevant, but AI virtual companion apps also require attention to emotional presence, personalization, memory, and relational continuity. The present model therefore explains acceptance as a process in which emotional design shapes user experience and, through that experience, behavioral intention.

The second contribution is the clarification of AI virtual companions as a distinctive media psychology context. AI companions are not merely tools for performing tasks. They invite users to engage in emotionally framed, repeated, and sometimes intimate interaction. This makes them closer to relational media than to ordinary software services. The finding that intelligent adaptability and perceived personalization are strongly associated with behavioral intention suggests that users value design features that make the system appear responsive to their particular emotional and interactional history.

The third contribution concerns the extension of emotional design theory to AI-mediated interaction. Norman’s visceral, behavioral, and reflective levels remain useful, but AI companion apps require an additional explanation of how algorithmic capabilities become emotionally meaningful. Intelligent adaptability is therefore theorized not as a separate emotion, but as an AI capability that supports behavioral smoothness and reflective personal meaning. This interpretation is consistent with recent work on digital humanism and AI, which emphasizes the role of emotion beyond narrow human–machine interaction (Magni et al., 2024), and with broader disruptive-technology research showing that the adoption of novel AI systems depends on how users and organizations absorb and interpret technological change (Gentile et al., 2007).

### 6.2 The impact of emotional design on user experience

First, the findings demonstrated that emotional design had a significantly positive influence on user experience, supporting H1. Among the emotional design dimensions, intelligent adaptability showed the strongest correlation with behavioral intention, followed by perceived personalization. This suggests that users may evaluate AI virtual companions less as static interfaces and more as systems that should learn, remember, and respond in context. The result is consistent with prior research on virtual companionship and relational continuity, which indicates that adaptive responsiveness can strengthen perceived emotional credibility and sustained engagement (Strohmann et al., 2023).

In practical design terms, the “intelligence” of AI virtual companions should not be understood only as computational accuracy. It becomes meaningful for users when it is translated into context-sensitive interaction, appropriate emotional feedback, and transparent personalization. At the same time, adaptive functions must be designed cautiously because inaccurate memory, opaque data use, or inappropriate emotional inference may undermine trust and make users feel misunderstood (De Freitas et al., 2024).

Visual appeal also remains important because it shapes users’ first impressions and affective readiness to interact. Appearance design, interface aesthetics, character style, and visual effects can make the app feel approachable and emotionally compatible. Nevertheless, visual appeal should be interpreted as an entry condition rather than the sole driver of sustained use. Long-term acceptance appears to depend more strongly on whether the system can provide coherent interaction, personalization, and adaptive responsiveness.

Overall, interaction quality, visual appeal, perceived personalization, and intelligent adaptability jointly influenced user experience. These dimensions should be understood as complementary rather than isolated design factors: visual appeal may attract initial attention, interaction quality reduces friction, personalization creates a sense of individual relevance, and intelligent adaptability supports continuity across repeated interactions.

### 6.3 The impact of user experience on behavioral intention

As data analysis unveiled, user experience greatly influenced single young adults’ behavioral intentions toward AI virtual companions, which supported H3. In detail, users’ emotional experiences and action experiences posed the most notable impact on behavioral intention. Especially, emotional experience (*β* = 0.364, *p* < 0.001) played a core role in users’ judgment of their intentions to continue using AI virtual companions. Similarly, the study by Savic (2024) evidenced that emotional experience influenced users’ long-term usage intentions decisively.

The influence of emotional experience indicates that AI virtual companions are evaluated partly through feelings of comfort, recognition, and conversational ease. However, these results should not be interpreted as demonstrating that AI companions clinically reduce loneliness or improve mental health. The data show associations between users’ reported experiences and behavioral intentions within a cross-sectional survey. Future longitudinal or experimental studies would be needed to determine whether such apps produce measurable psychological benefits over time.

Moreover, action experience (*β* = 0.301, *p* < 0.001) was closely tied to users’ interaction enthusiasms and involvement degrees. Whether users intended to interact with more time and effort or not directly affected their behavioral intentions. Therefore, when developing AI virtual companions, designers should not only focus on emotional design but also guide users to participate in daily activities and tasks through interaction design to boost users’ use frequencies.

### 6.4 The impact of emotional design on behavioral intention

Regarding H2, the data confirmed that emotional design had a positive direct effect on behavioral intention (*β* = 0.377, *p* < 0.001). This implies that emotional design can influence acceptance before or alongside the formation of detailed user experience. Users may form early judgments about whether the companion app appears warm, trustworthy, and personally relevant. This finding aligns with technology acceptance research showing that behavioral intention can be shaped by both instrumental and affective evaluations (Chaves and Gerosa, 2021).

At the same time, the mediation result indicates that emotional design is particularly consequential when it becomes part of the user’s lived experience. This is important for AI virtual companions because users are unlikely to continue using a system merely because it has advanced functions. They must experience those functions as coherent, emotionally appropriate, and personally meaningful.

### 6.5 The mediating role of user experience

Regarding H4, this research investigated whether user experience mediated between emotional design and behavioral intention or not. The data analysis results supported this hypothesis (indirect effect = 0.139, 95% CI: 0.098–0.188, *p* < 0.001). Namely, user experience partially mediated between emotional design and behavioral intention, reflecting that the dimensions of emotional design affected users’ behavioral intentions through user experience optimization. Just as confirmed by Bassi et al. (2021), user experience bridged between emotional design and behavioral intention.

More in-depth analysis revealed that emotional and relational experiences held important positions within this mediating effect. Emotional experience can strengthen intention by making users feel recognized and comforted, while relational experience can make interaction feel more continuous and socially meaningful. Nevertheless, relational experience should be designed with safeguards. Companion apps should avoid encouraging excessive dependence, should provide transparent privacy controls, and should direct users toward human or professional support when users express severe distress.

Taken together, the hypotheses show that emotional design influences single young adults’ acceptance intention both directly and indirectly through user experience. The findings support the view that AI companion acceptance is not merely a matter of technology performance, but a media psychology process involving emotional design, perceived relationality, trust, and cultural interpretation.

### 6.6 Implications for AI virtual companions and human companionship

As human–AI emotional interaction develops, AI virtual companions should be positioned as supplementary digital companions rather than replacements for human relationships. The present findings indicate that emotional design features are associated with acceptance intention among current users, but they do not prove that AI companions can replace human empathy, solve loneliness at the societal level, or reshape intimate relationships in a deterministic way. A more responsible interpretation is that AI companions may provide low-threshold conversation, routine emotional support, or fragmented-time interaction for some users, while human relationships and professional support remain irreplaceable in complex emotional, ethical, and social situations (Table 27).

**Table 27:** Conceptual comparison of AI virtual companions and human companionship under a supplementary-use perspective.

Dimension	AI virtual companions: potential benefits and risks	Human companionship: distinctive strengths and limits	Supplementary-use design implications
Accessibility	24/7 online; globally accessible/relying on device net	Co-presence interaction; non-verbal feedback/having temporal and spatial limitations	Designing progressive emotional trials and supplementing fragmented periods
Emotional response	Fast learning; stable emotion recognition/rational algorithmized empathy	Complex emotions; genuine empathy/emotional fluctuations	Launching “human-AI emotional coaching” dual-track services industrially
Privacy security	Allowing for encrypting records but having data leakage risks	“Instant disappearance” upon communication but having personal risks	Formulating policies on “digital intimacy rights” to provide tiered open guidelines
Growth and learning	Continuous algorithm evolution/data bias	Growing together; rich experience/limited cognition	Exploring longitudinal cohort tracking for feedback-driven algorithmic fairness
Social acceptance	Emerging phenomenon; stigma and regulatory blank	Cultural mainstream; legal support	Setting up “digital intimate relationship” courses to eliminate stigma
Cost-effectiveness	Low marginal costs; with subscription/upgrade fees	Hidden monetary costs; high emotional investment/economic pressure	Combining design with industry for emotional subscription to reclaim high-value time for real human connections

In sum, AI virtual companions and human companions should not be framed as a zero-sum relationship. The design challenge is to create systems that can supplement users’ daily emotional experiences without encouraging withdrawal from human relationships, undermining privacy, or exploiting emotional vulnerability. Future product development should therefore combine personalization, transparency, safety reminders, and culturally sensitive communication strategies.

## 7 Conclusion and future directions

Grounded in emotional design theory, this research explored the factors influencing single young adults’ intentions to accept AI virtual companions. By integrating emotional design, technology acceptance, human–AI interaction, and digital companionship perspectives, the study examined how interaction quality, visual appeal, perceived personalization, and intelligent adaptability shape user experience and behavioral intention. The empirical results supported the proposed SEM model and showed that user experience partially mediated the relationship between emotional design and behavioral intention.

### 7.1 Main findings

The main conclusions of this research can be summarized as follows.

#### 7.1.1 The significant impact of emotional design on user experience

This research revealed that emotional design dimensions, particularly intelligent adaptability, perceived personalization, interaction quality, and visual appeal, were positively associated with user experience and behavioral intention. Intelligent adaptability was especially important because it allowed users to perceive the companion as capable of learning, remembering, and responding to their needs. However, this finding should be interpreted as a design-related acceptance result, not as evidence that adaptive AI companionship is inherently beneficial in all situations.

Visual appeal and interaction quality were also crucial for enhancing user experience. Visual appeal influenced users’ initial involvement, while interaction quality affected the fluency, naturalness, and emotional appropriateness of communication. These findings suggest that effective AI companion design requires both an accessible interface and a credible interaction process.

#### 7.1.2 The mediating role of user experience between emotional design and behavioral intention

The research showed that user experience mediated the relationship between emotional design and behavioral intention. Specifically, emotional, action, and relational experiences helped translate design features into acceptance intention. This result highlights that users do not simply respond to isolated functions; they respond to the quality and meaning of the interaction experience created by those functions.

The mediating effect further underscores the theoretical value of connecting emotional design with user experience. Emotional design can influence immediate impressions, but sustained acceptance depends on whether users experience the system as smooth, attentive, personalized, and trustworthy during actual interaction.

#### 7.1.3 The direct impact of emotional design on behavioral intention

The research also found that emotional design had a direct positive influence on behavioral intention. Design factors such as intelligent adaptability, visual appeal, and perceived personalization can directly affect whether single young adults are willing to accept, continue using, or recommend AI virtual companion apps. This result supports the relevance of emotional design theory for media psychology and human–AI interaction research.

### 7.2 Recommendations

Based on the above conclusions, the following recommendations are proposed.

#### 7.2.1 Enhancing intelligent adaptability and personalized design

Because intelligent adaptability was a key factor associated with user experience and behavioral intention, AI virtual companion design should place greater emphasis on transparent and controllable personalization. Designers can allow users to decide what information the system may remember, how adaptive responses are generated, and when personalization can be reset or disabled. Such transparency can help ensure that adaptive design strengthens trust rather than creating privacy concerns or feelings of manipulation (Merrill et al., 2022).

#### 7.2.2 Optimizing visual appeal and interaction quality

Since visual appeal affects initial experience, designers should create companion images and interfaces that align with the aesthetic preferences of the target users while avoiding homogenization and uncanny-valley effects. Interaction quality should be improved through natural language understanding, response coherence, appropriate response speed, and emotionally sensitive expression. These features can help the app appear approachable without overstating its human-like qualities (Chan, 2025).

#### 7.2.3 Improving emotional experience and action experience

Because user experience was positively associated with behavioral intention, designers should focus on emotional resonance, relational continuity, and meaningful engagement. Nevertheless, design should avoid implying that AI companions can replace human relationships or solve loneliness on their own. Appropriate safeguards may include privacy reminders, emotional-dependency warnings, crisis-support links, and design cues that encourage users to maintain offline social relationships when needed (Liu et al., 2026).

#### 7.2.4 Research limitations and future research directions

Although this research offers theoretical and practical references for the design of AI virtual companions, it has several limitations. First, the study focused exclusively on Chinese single young adults aged 18–35 and used online recruitment with snowball sampling. The findings therefore should not be generalized to all young adults, all single adults, or users in other cultural contexts. Cultural norms concerning intimacy, privacy, stigma, gender roles, and emotional disclosure may substantially influence perceptions of AI companionship and emotional design. Future studies should compare different cultural settings and include participants with more diverse demographic backgrounds.

Second, the research used cross-sectional survey data. Although SEM is useful for testing theoretically specified relationships, it cannot establish causal effects or reveal how acceptance changes over time. Longitudinal studies, diary studies, experiments, and behavioral log analyses are needed to examine whether emotional design has sustained effects on continued use, emotional attachment, dependency risk, or wellbeing outcomes.

Third, all constructs were measured through self-report items. Although reliability, convergent validity, discriminant validity, and common method bias checks supported the measurement model, future research could combine self-report data with interviews, usage logs, physiological indicators, or experimental tasks. In addition, future work should further investigate ethical variables such as privacy concern, trust, perceived manipulation, emotional dependence, and willingness to seek human support.

In sum, this research investigated how emotional design factors of AI virtual companions influence the acceptance intentions of single young adults. The results showed that emotional design, especially intelligent adaptability and perceived personalization, is positively associated with user experience and behavioral intention, and that user experience partially mediates this relationship. These findings provide theoretical support and practical guidance for designing AI virtual companion apps that are more responsive, transparent, and user-sensitive. At the same time, the conclusions should be understood within the study’s cultural, sampling, and cross-sectional boundaries, and future research should examine long-term outcomes and cross-cultural differences.

## Data Availability

The original contributions presented in the study are included in the article/supplementary material, further inquiries can be directed to the corresponding author.
